# High Pressure Torsion of Copper; Effect of Processing Temperature on Structural Features, Microhardness and Electric Conductivity

**DOI:** 10.3390/ma16072738

**Published:** 2023-03-29

**Authors:** Lenka Kunčická, Michal Jambor, Petr Král

**Affiliations:** Institute of Physics of Materials, Czech Academy of Sciences, Žižkova 22, 616 00 Brno, Czech Republic

**Keywords:** copper, high pressure torsion, microstructure, electric conductivity, twinning

## Abstract

By optimizing the fabrication method, copper components featuring (typically contradicting) advantageous electric conductivity and favorable mechanical properties can be acquired. In this study, we subjected conventional electroconductive copper to a single revolution of high pressure torsion (HPT) at room temperature (RT), searched for the conditions which would yield comparable structure characteristics (grain size) when deformed at a cryogenic temperature, and finally compared the mechanical and electric behaviors to assess specific differences and correlate them with the (sub)structural development. 180° revolution of cryo-HPT imparted structure refinement comparable to 360° revolution of room temperature HPT, i.e., the average grain size at the periphery of both the specimens was ~7 µm. The 360° RT HPT specimen exhibited preferential (111)||SD (shear direction) texture fiber in all the examined regions, whereas the 180° cryo-HPT specimen exhibited more or less randomly oriented grains of equiaxed shapes featuring substantial substructure development of a relatively homogeneous character and massive occurrence of (nano)twins. These structural features resulted in the increase in microhardness to the average value of 118.2 HV0.2 and the increase in the electric conductivity to 59.66 MS·m^−1^ (compared to 105 HV0.2 and 59.14 MS·m^−1^ acquired for the 360° RT HPT specimen). The deformation under the cryogenic conditions also imparted higher homogeneity of microhardness distribution when compared to RT processing.

## 1. Introduction

Copper features very high electric conductivity, and it is even the standard for excellent electric conductivity worldwide (electric conductivity is often determined according to the IACS, i.e., International Annealed Copper Standard). Nevertheless, excellent conductivity is generally only observed for Cu of a high purity, which is typically produced by electrolytic refining, enabling it to achieve a purity of up to 99.99% [1]. Any impurities within the structure, especially Fe, Si, As and O, tend to aggravate the movement of free electrons and thus deteriorate the electric conductivity. The yield strength of such a highly pure Cu is only about 10–20 MPa. Additional and alloying elements introduce strengthening, primarily via solid solution and formation of precipitates, which increase the mechanical properties. For example, minor amounts of Cd or Ag can be added to enhance the mechanical properties and performance of Cu at elevated temperatures. However, improvements in the mechanical properties are typically at the expense of electric conductivity [2,3,4].

Among the possibilities how to increase the mechanical properties and simultaneously maintain advantageous electric conductivity is to use modern methods and fabricate Cu-based composites featuring strengthening elements (e.g., powder-based composites with additions of Cr [5], Al_2_O_3_ [6], and carbon nanotubes [7], or clad composites consisting of Cu plus Al [8,9,10,11], Al and Mg [12], Nb [13], and FeSiBCuNb [14]). A favorable way how to increase the mechanical properties without deteriorating the electric ones can also be optimized deformation (thermomechanical) treatment–such as fabrication of electro-conductive wires via rotary swaging [15] or processing via methods of severe plastic deformation (SPD) [16]. Such methods can be used to strengthen the processed materials by imparting generation of dislocations, formation of dislocation cells and walls, and polygonization, i.e., formation of subgrains, which finally develop into fully refined grains [17]. Hansen et al. [18] documented that the spacing between deformation-induced boundaries and their misorientation angles are the key parameters controlling the grain refinement and consequent strengthening of severely deformed materials. Their flow stress is thus primarily affected by dislocation strengthening related to the occurrence of low-angle grain boundaries and Hall–Petch strengthening related to the presence of medium to high-angle grain boundaries. However, the ratio of these two affecting parameters strongly depends on the imposed strain and other material factors, such as grain orientations [19].

Amongst the most widely applied SPD methods is Equal Channel Angular Pressing (ECAP) [20] and its modifications (twist channel (multi)angular pressing [21,22], non-ECAP [23], ECAP with partial back pressure [24], etc.). The positive effects of ECAP on structure refinement and mechanical behavior of commercially pure Cu were documented, e.g., by Volokitina [25] or Hlaváč et al. [26], whereas, for example, Guo et al. [27] and Huang et al. [28] performed studies on ECAP-deformed Cu-based alloys. ECAP-based methods have the advantage that they can be used to process bulk samples of relatively large volumes (considering the range of the SPD methods). Among the efficient SPD methods which enable the processing of larger volumes of materials is the Accumulative Roll Bonding (ARB) method, which is based on conventional rolling and thus also industrially applicable [29]. Tayyebi et al. [12] involved ARB in an optimized thermomechanical treatment and fabricated layered Al/Cu/Mg composites. ARB was also applied to fabricate clad composites of optimized properties by Rahmatabadi et al. [30]. Nevertheless, there are SPD methods that are considered to be more efficient than ECAP or ARB as regards grain refinement–primarily friction stir processing (FSP) [31] and high-pressure torsion (HPT) [32].

HPT was probably the first ever introduced SPD method and has had several basic modifications as regards the dimensions of the processed specimen and the geometry of the dies [33]. Nevertheless, the basis of the method is that a round specimen is deformed between two massive anvils under very high pressure (typically up to 5 or 6 GPa). The combination of high pressure and severe shear strain is especially advantageous–the primary deformation mechanism during HPT is simple shear [34]. When a high shear strain is imposed into the material, the deformation tends to localize in shear bands, which progressively (if deformation continues) increases the deformation inhomogeneity. However, the application of high pressure during the deformation contributes to increasing the shear strain homogeneity. For this reason, HPT typically imparts massive substructure development and grain fragmentation throughout the deformed specimen (depending on the imposed strain). Studies investigating the effects of HPT on (sub)structure development within commercially pure Cu can be found, e.g., An et al. [35] observed the effects of stacking fault energy of Cu and selected Cu-based alloys during HPT processing, and Huang et al. [36] and Schafler and Kerber [37] studied the behaviors of HPT-processed Cu after annealing at room and elevated temperatures for relatively long time periods. Nevertheless, as far as the authors’ knowledge reaches, there is no (comparative) study investigating the influence of HPT on the electric behavior of Cu. Neither is there any study comparing the effects of HPT processing at room temperature and cryogenic temperature.

The study presents the results of the processing of electro-conductive commercially pure Cu via HPT at room temperature and at cryogenic conditions. The hypothesis was that structures with comparable characteristics (e.g., grain size) but acquired under different conditions feature specific differences, which consequently introduce differences in the mechanical and electric properties. The core idea beyond this experiment was thus to find out the conditions of deformation at room and cryogenic temperatures, which would yield comparable results as regards (sub)structure development within the original Cu, and then compare the processed specimens from the viewpoints of the mechanical properties, i.e., microhardness, and electric properties. In order to achieve the mentioned goal, we performed a single revolution, i.e., rotation by 360°, HPT at room temperature, and then assessed the efficiency of cryogenic processing by looking for comparable structure states, primarily as regards the grain size. In other words, we assessed the effects of one revolution of room temperature HPT on (sub)structure, microhardness, and electric conductivity of the original Cu and then compared the results with those acquired for specimens deformed by 180°and 360° HPT rotation at a cryogenic temperature.

## 2. Materials and Methods

As for the initial material, we used commercially available electro-conductive Cu of commercial purity (CP), with impurities of 0.015 wt.% P, 0.002 wt.% Zn, and 0.002 wt.% O. At first, we applied a heat treatment–600 °C for 30 min–to relax the structure. Subsequently, we cut the annealed billet into pieces to acquire HPT specimens with the initial dimensions of 20 mm in diameter × 6 mm in height. The specimens were deformed at the pressure of 3 GPa with the rotation speed of 1 RPM, either at room or cryogenic temperature (cooled by liquid nitrogen). In accordance with the aim of the study, we performed a single HPT revolution (360° rotation) for the room temperature specimen (denoted as RT360) and a single HPT revolution (360° rotation) and half revolution (180° rotation) for the specimens cooled to the cryogenic temperature (denoted as CR360 and CR180).

**Figure 1:**
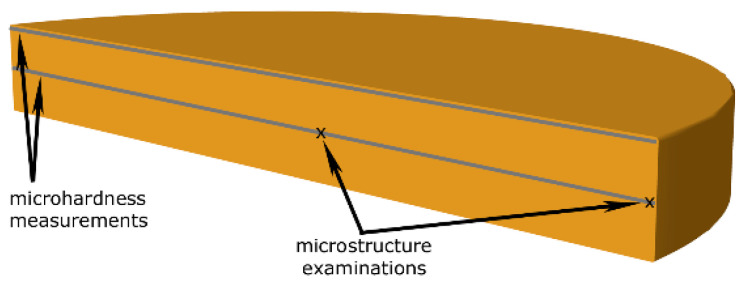
Schematic depiction of analyzed locations.

After processing, scanning electron microscopy (SEM) structure analyses were carried out using the Tescan Lyra 3 XMU FEG/SEMxFIB device (Tescan Orsay Holding a.s., Brno, Czech Republic). To acquire the structure scans, which we subsequently evaluated with the help of the AZtecCrystal software (version 2.2), we used the electron backscatter diffraction feature for which we used the Symmetry EBSD detector equipped with the AZtec software (both detector and software by Oxford Instruments, Abingdon, UK). To prepare the EBSD samples, the HPT specimens were cut transversally to expose their cross-sectional planes (see Figure 1 for the depiction of the locations of the performed analyses). The samples were then manually ground and polished using diamond solutions with the coarseness down to 0.6 µm and finally polished electrolytically (solutions by Struers GmbH, Roztoky u Prahy, Czech Republic). The 100 × 100 µm^2^ EBSD scans were acquired with the step of 0.1 µm (for the analyses of the original coarse-grained CP Cu, the scanned area was 500 × 350 µm^2^). To evaluate the results, the following limits were used: LAGBs (low angle grain boundaries) 5–15°, HAGBs (high angle grain boundaries) > 15°.

Having performed the structure evaluations, we compared the results acquired from the RT and CR specimens and selected the CR specimen that featured structure characteristics comparable to those observed for the RT360 specimen. The following analyses were thus further performed only for the two selected specimens featuring comparable structural features.

In order to assess the structure development in detail and compare substructural features, detailed substructure analyses of both the selected specimens were performed using the JEM-2100 transmission electron microscope (TEM, JEOL, Tokyo, Japan) operating at 200 kV. The foils for the analyses were manually ground and finally electrolytically polished using the LectroPol-5 device (Struers GmbH, Roztoky u Prahy, Czech Republic).

To evaluate the electric characteristics of the processed specimens, we used the SIGMATEST 2.070 device (FOERSTER TECOM s.r.o, Prague, Czech Republic). This high-tech eddy current portable device measures the electric conductivity of non-ferromagnetic materials based on the complex impedance of the measuring probe. This principle is advantageous, especially for determining the electric characteristics of specimens of small dimensions, such as the ones presented herein. The device was at first calibrated using two default specimens with different electro-conductivities. Subsequently, the measurements of the electric characteristics of the HPT-processed specimens could be performed by connecting the probe of the device to the measured specimen. The measured value of electroconductivity (both in MS·m^−1^ and % IACS) for each specimen was then shown directly on the screen of the device.

Finally, Vickers microhardness HV0.2 (load of 200 g) along two lines across the cross-sections of the specimens (at the periphery of the specimen, as well as along its axial line, see Figure 1) was measured using a Zwick Roell DuraScan 70 G5 device (Zwick Roell CZ s.r.o., Brno, Czech Republic). The load time for each indent was 10 s.

## 3. Results

### 3.1. Grains Evaluation

**Figure 2:**
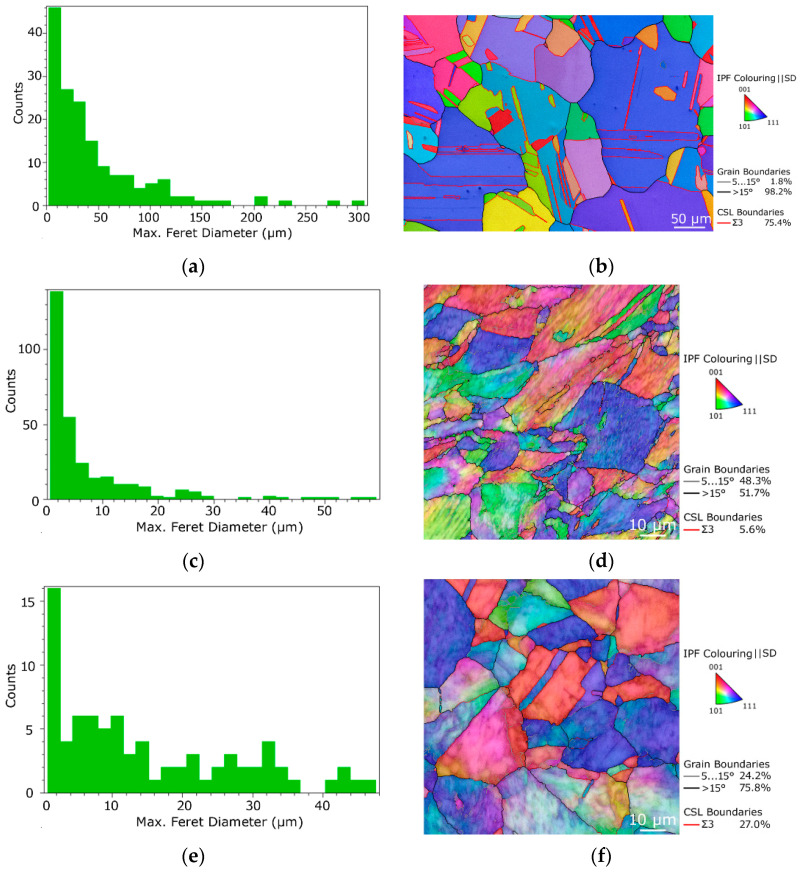

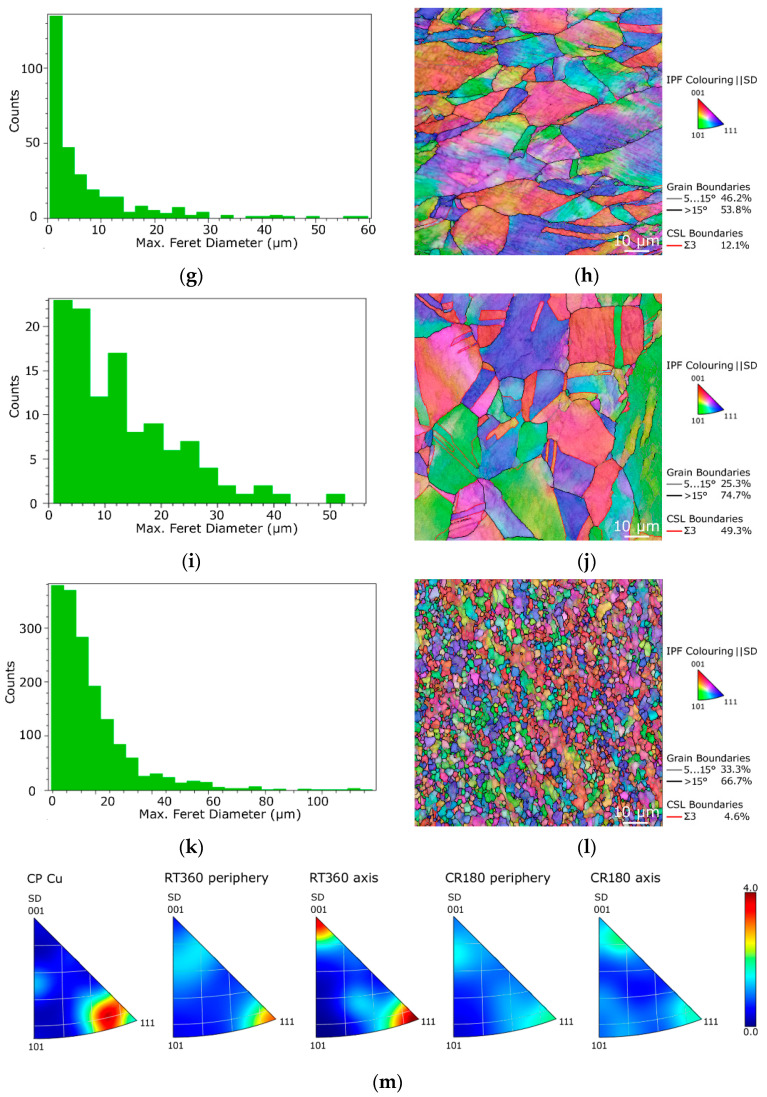
Grain size charts and OIM images, respectively, for: original Cu (**a**,**b**); RT360 specimen periphery (**c**,**d**); RT360 specimen axis (**e**,**f**); CR180 specimen periphery (**g**,**h**); CR180 specimen axis (**i**,**j**); CR360 specimen periphery (**k**,**l**). IPF images showing intensities (max. 4× random for all IPFs) of <111>||SD texture fiber for CP Cu and regions of RT360 and CR180 specimens (**m**).

The grains within the CP Cu, as well as deformed specimens, were examined from the viewpoints of their size, morphology, and orientations. Figure 2a shows the distribution of grain size characterized via the max. feret diameter in µm for CP Cu, while Figure 2b shows the orientation image map (OIM) for the CP Cu structure with depicted LAGBs, HAGBs, and <111>60° twin boundaries. The grains within the original material featured an average size of 46.1 µm; however, the largest grains reached almost 300 µm. The grain boundaries within the structure were primarily the HAGBs, among which prevailed the <111>60° twin boundaries.

The grain size charts and OIM images for the peripheral and axial regions of the RT360 specimen are depicted in Figure 2c–f. According to the structure observations, the grain size refined significantly after the single revolution of room temperature HPT, and the avg. grain size in the axial region of the specimen was 14.8 µm, while in the peripheral region, it was as low as 7.1 µm. Obvious differences were, however, in the characters of the structures, i.e., grains morphology. The peripheral region of the specimen exhibited fragmented and visibly sheared grains with well-developed substructure and a high fraction of LAGBs (almost 50%). The grains in the axial region of the specimen were fragmented, too, but their shapes were more equiaxed and without visible traces of shearing. The structure within this region also featured substructure development (see the color shading within individual grains in Figure 2f), but a greater portion of twins when compared to the peripheral region. The grains in both the regions of the RT360 specimen exhibited a tendency to form the <111>||SD (shear direction) texture fiber (Inverse Pole Figures–IPF–for the examined regions of the investigated specimens are shown in Figure 2m to enable mutual comparison of the maximum intensities of the <111>||SD fiber).

Figure 2g–j depicts the grain size charts and OIM images for the peripheral and axial regions of the CR180 specimen. Evidently, this specimen featured comparable grain sizes to the RT360 one; the avg. grain size in the peripheral region of the specimen was 7.2 µm. The grain size in the axial region was slightly lower than within the RT360 specimen, i.e., 13.3 µm. The difference between the grain size values acquired in the peripheral and axial regions of the CR180 specimen was smaller than for the RT360 specimen. In other words, the structure homogeneity was slightly higher for the CR180 specimen. The structures in the axial regions of both specimens were comparable as regards the shapes of the grains. However, the CR180 specimen featured a greater portion of twin boundaries (compare Figure 2f,j). Compared to the RT360 specimen, the grains at the periphery of the CR180 one were also fragmented and featured a similar fraction of LAGBs, but exhibited less sheared shapes and featured a greater portion of twin boundaries. As regards the orientations of the grains, they also exhibited a tendency to form the <111> || SD texture fiber, but with a lower intensity than the RT360 specimen (Figure 2m).

The structure acquired at the periphery of the CR360 specimen, together with the corresponding grain size chart, are depicted in Figure 2k,l, respectively. One HPT revolution at a cryogenic temperature evidently led to massive fragmentation of the grains, as the structure featured fine equiaxed grains with the avg. diameter of 3.6 µm. According to the structure observations, the specimen deformed by 180° rotation at a cryogenic temperature featured structure characteristics comparable to those acquired after 360° rotation at room temperature. Based on this finding, the following detailed investigations of substructure, microhardness, and electric properties focused on examining specific differences imparted by the selected processing temperature were performed for the specimens featuring comparable structures, i.e., RT360 vs. CR180.

### 3.2. Substructure

**Figure 3:**
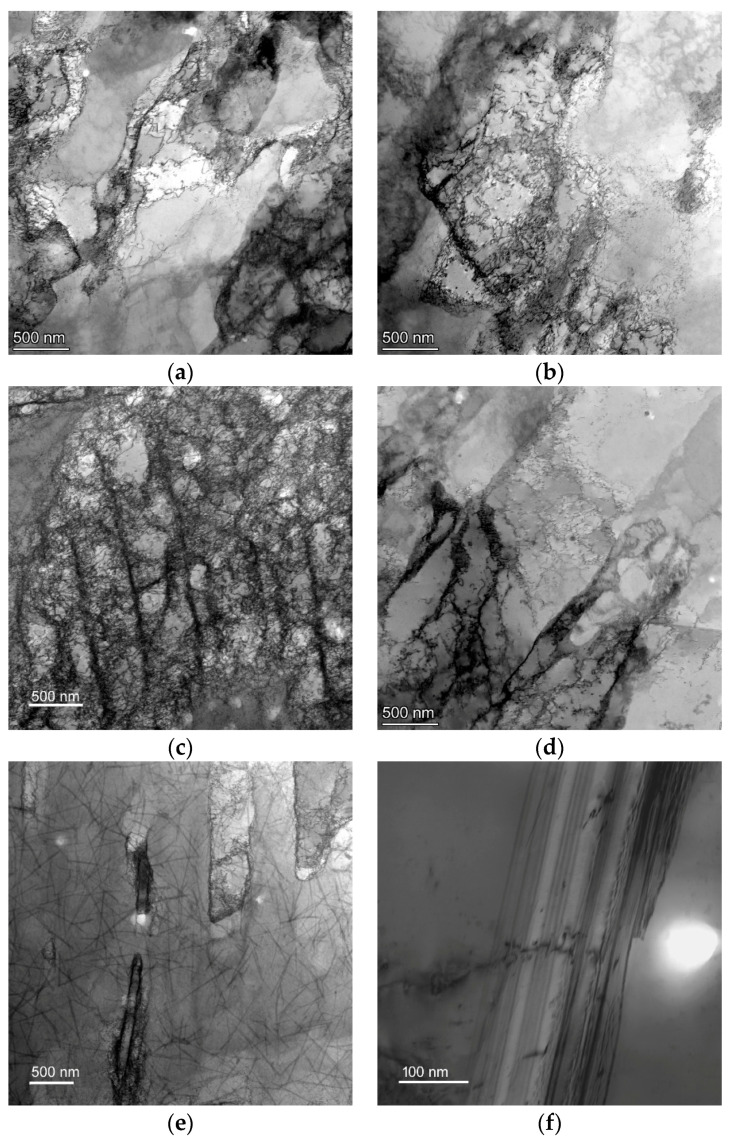
TEM substructure images for RT360 specimen periphery (**a**); RT360 specimen axis (**b**); CR180 specimen periphery (**c**); CR180 specimen axis (**d**); (nano)twinning within the CR180 specimen (**e**,**f**).

The above characterized SEM observations revealed that the RT360 and CR180 specimens featured comparable microstructures. However, characteristic differences could be observed as regards the substructure development. TEM substructure images acquired from the peripheral and axial regions of the RT360 specimen are depicted in Figure 3a,b, whereas the substructures acquired from the peripheral and axial regions of the CR180 specimen are shown in Figure 3c,d, respectively. Evident differences are visible, especially at the peripheries; the CR180 specimen obviously featured a higher level of substructure development compared to the RT360 one (compare Figure 3c to Figure 3a). In other words, the dislocation density, as well as the occurrence of dislocation cells and dislocation walls, was higher within the CR180 specimen. As regards the axial regions, both specimens exhibited similarities from the viewpoint of dislocation substructure. Nevertheless, the CR180 specimen featured the occurrence of twins and especially nanotwins, which was not observed within the RT360 specimen. These characteristic features are depicted in Figure 3e,f.

### 3.3. Electric Conductivity

**Figure 4:**
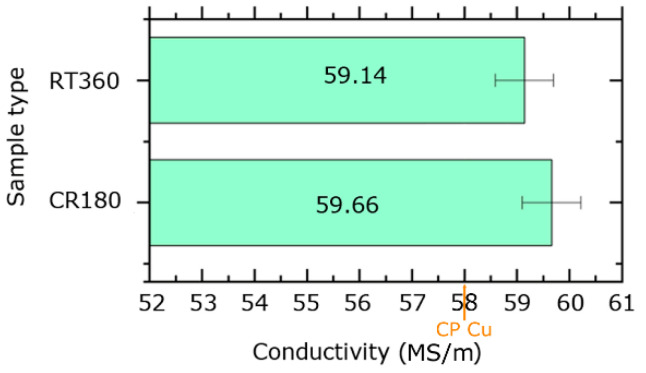
Depiction of experimentally measured electric conductivity for RT360 and CR180 specimens with comparison to CP Cu.

The experimental measurements of the electric conductivity were performed ten times for each specimen in order to also evaluate the standard deviation from the average value. The measured values were compared with the standard electric conductivity for Cu, i.e., the value measured for the original annealed CP Cu of σ_Cu_ = 58 MS·m^−1^. The results are graphically depicted in Figure 4. Evidently, compared to the CP Cu, the electric conductivity increased for both the HPT-processed specimens. The CR180 specimen exhibited even higher conductivity than the RT360 one.

### 3.4. Mechanical Properties

**Figure 5:**
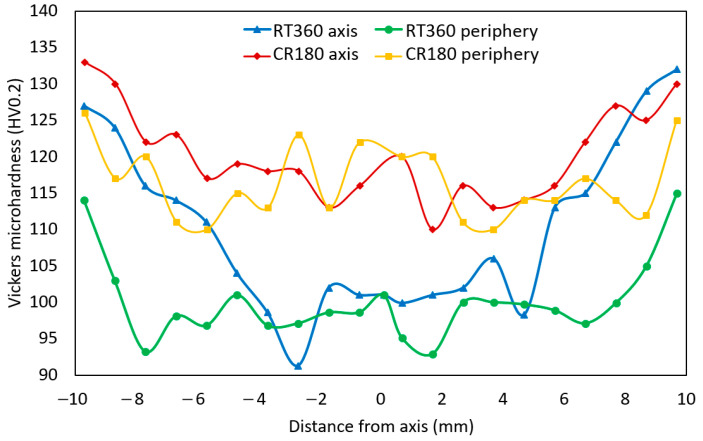
Vickers microhardness across deformed specimens.

The results of measurements of HV0.2 Vickers microhardness for both the HPT-processed specimens are depicted in Figure 5, showing the values acquired along two characteristic lines across the cross-sections of the examined specimens (see Figure 1). The original CP Cu featured an average microhardness of 43.6 HV0.2. As can be seen from the Figure, the values remarkably increased after processing at both the room and cryogenic temperatures. The average microhardness values for the axial and peripheral regions of the RT360 specimen were 100.1 HV0.2 and 109.9 HV0.2, while the values for the CR180 specimen were 116.4 HV0.2 and 120.1 HV0.2, respectively. Evidently, the processing via 180° rotation at a cryogenic temperature led to a higher increase in microhardness when compared to the processing by 360° rotation at room temperature (compare the CR180 curves to the RT360 ones in Figure 5), and it also imparted increased homogeneity of microhardness values throughout the specimen (the standard deviation for the RT360 specimen was 10.2, while for the CR180 one, it was 5.9). As regards the RT360 specimen, its peripheral region exhibited the highest HV0.2 values, which then gradually decreased towards the axial region. The CR180 specimen, on the other hand, featured greater microhardness homogeneity as the range of the HV0.2 values measured in both the axial and peripheral regions was more or less comparable.

## 4. Discussion

The structures within both the examined regions of the RT360 specimen featured differences, especially as regards the development of the substructure. As the imposed strain for the HPT technology is the greatest at the periphery of the processed specimen and decreases towards its axis [33], the level of (sub)structure development is, correspondingly, expected to be the highest in the peripheral region of the specimen and to decrease gradually towards its axis. Such development was also observed within the RT360 specimen, as documented by the herein presented Figure 2d,f. The periphery of the specimen featured fragmented grains, some of which were with no evident substructure development. Most of the grains in the axial region, on the other hand, exhibited evident shading, which points to substantial substructure development. This finding corresponds with the relatively large fraction of LAGBs observed in this region, too. Considering also the fact that the average grain size was approx. two times higher in the axial region than at the periphery of the RT360 specimen, the following hypothesis can be given. The severe shear strain imposed during HPT processing promoted the occurrence of the chain of the following structure-forming phenomena: grain fragmentation-substructure development-nucleation of new grains. However, as the shear strain imposed on the structure in the peripheral region of the RT360 specimen was very high, the mentioned chain of structure-forming processes in this region proceeded to a greater extent than in the axial region. In other words, the structure observed at the peripheral region of the RT360 specimen featured refined grains, which underwent a higher number of repetitions of grains fragmentation and restoration, whereas the structure observed at the axial region of the specimen featured grains with developed substructure, which were about to fragment and refine again (if deformation continued). This hypothesis was supported by the TEM observations. The scan in Figure 3a acquired from the peripheral region of the RT360 specimen featured not only locations exhibiting the presence of dislocation cells and walls with a high density of dislocations but also restored grains with subgrains with very low dislocations density. On the other hand, the scan acquired from the axial region of the specimen featured evidently larger grains with developed dislocations substructure.

Deforming the specimen at a cryogenic temperature imparted (sub)structure development, too, but with specific differences. Among the most significant differences between the structures of the RT360 and CR180 HPT-processed specimens is the mechanism of grain fragmentation. As the shear strain during HPT is very high and, moreover, is imposed under very high pressures, the development of internal heat, i.e., a certain increase in temperature during processing, is expected to occur (up to 90% of the energy imposed to the material during plastic deformation can be consumed by the development of intrinsic deformation heat [38]). Therefore, the mobility of dislocations during room temperature deformation via shear strain is expected to increase with continuing deformation by the effect of thermally activated dislocation motion [39]. At the temperature of 0 K, no thermal fluctuations exist, and plastic flow can occur only if the applied shear stress *τ* is greater than the critical shear stress *τ_0_*. However, with an increase in temperature, the applied shear stress necessary to activate the deformation processes decreases, i.e., *τ < τ_0_*. In accordance with this statement, the mobility of atoms and dislocations was hindered during HPT deformation at a cryogenic temperature. For this reason, the grains within the CR180 specimen were not visibly sheared to such an extent as within the RT360 specimen–they preferably deformed by twinning, which is a less demanding deformation mechanism than shear sliding. This hypothesis is also supported by the fact that Cu is a metal with relatively low stacking fault energy (SFE), and thus decreasing the processing temperature aggravates the activity of slip systems and supports twinning [1].

The presence of this structural phenomenon, i.e., twinning, and the extent to which it occurred was another difference between the CR180 and RT360 specimens. A twin subdivides the grain and reorients a portion of its crystal lattice. Therefore, it introduces new possibilities for the dislocations to slip due to the reorientation (of a portion) of the slip planes to new (more advantageous) positions [40,41]. By the effect of these structure-forming phenomena, the grains within the CR180 specimen were of more or less equiaxed shapes than visibly deformed shapes. The fact that it was impossible to determine the exact moment at which a twin developed should be mentioned. In other words, annealing twins (remnants of the original structure of the CP Cu) and deformation twins can hardly be distinguished. On the other hand, the grains featuring the twins within the CR180 specimen were far smaller than within the original CP Cu. This fact gives rise to the supposition that the observed twins were deformation twins developed due to the aggravated mobility of atoms within the crystal lattices of Cu and that twinning was a highly preferred deformation mechanism during the deformation at cryogenic conditions. This supposition was also supported by the TEM observations, which showed that twinning within the CR180 specimen occurred not only on the micro-scale but also on the nano-scale.

Murashkin et al. [42] presented the hypothesis that the presence of twin boundaries within the structure can improve the electric conductivity of Cu, as the development of twin boundaries tends to decrease the density of lattice defects at regular grain boundaries. Especially nano-scale twins with a twin spacing of ~15 nm were reported to enhance the conductivity, as the intrinsic grain boundary scattering is very low for them. Figure 3e, and especially Figure 3f, show the occurrence of multiple nano-scaled twins within the CR180 specimen. This feature was most probably among the factors favorably affecting the electric conductivity the most (the herein experimentally measured electric conductivity was the highest for the CR180 specimen). The SEM grain boundary analyses support this conclusion, too. The comparison of the boundaries observed within both the deformed specimens (Figure 2d,f,h,j) shows that the fractions of LAGBs and HAGBs were comparable for the individual regions of both the specimens (i.e., 48.3% and 46.2% of LAGBs in the peripheral regions of the RT360 and CR180 specimens, and 24.2% and 25.3% of LAGBs in the axial regions of the RT360 and CR180 specimens, respectively). However, the fractions of <111>60° twin boundaries differed remarkably, compared 5.6% to 12.1% in the peripheral regions of the RT360 and CR180 specimens and 27.0% to 49.3% in the axial regions of the RT360 and CR180 specimens, respectively. The more or less equiaxed shapes of the grains were advantageous, too. Typically, it is advantageous for the structures to feature optimized textures facilitating the movement of electrons during the transfer of the electric current [43]. However, the HPT specimens were of coin-like shapes, i.e., they were not of the wire-like shape of a conventional conductor. Nevertheless, not all electro-conductive applications require wire-like shapes of the conductors (e.g., microelectronics). Therefore, the absence of deformation texture is advantageous for smaller specimens since the anisotropy of electric properties can be avoided.

The observed (sub)structure development also resulted in a substantial increase in the mechanical properties, i.e., Vickers microhardness. The average HV0.2 increased for the RT360 specimen when compared to the CP Cu and increased even more for the CR180 one. As regards the values, the average microhardness increased from the original 43.6 HV0.2 to the average values of 105.0 HV0.2 for the RT360 and 118.2 HV0.2 for the CR180 specimens. In other words, the increase was by approx. 240% for the RT360 specimen and by more than 270% for the CR180 one. Also, the homogeneity of the values measured across the cross-section of the RT360 specimen was not as high as for the CR180 one. In other words, the specimen deformed at the cryogenic conditions featured a more homogeneous grain size distribution than the RT360 one, as well as greater development of substructure within the grains (TEM observations revealed the presence of dislocation substructure in the majority of the observed grains in the CR180 specimen, whereas the structure of the RT360 specimen featured also restored grains with very low dislocations density), both of which contributed to a more homogeneous distribution of HV0.2 values. Edalati et al. [44] reported the increase in microhardness after one HPT revolution to be up to the value of 132 HV. However, the microhardness of the original Cu used in their study was slightly higher than the herein presented (~50 HV). When compared to the work discussed herein in which we applied the pressure of 3 GPa, they applied a lower pressure of 2 GPa. This difference in pressures most probably introduced the difference in the structure development, i.e., supported twinning at the expense of dislocation slip (Edalati et al. did not report any significant occurrence of twins within the deformed structure). The occurrence of twins to a greater extent did not only contribute to the increased electric conductivity, as mentioned above but also to the increase in microhardness, as twinning within FCC metals has a very high work-hardening ability [1]. A slightly lower increase in microhardness (increment in percentage compared to the herein presented) for a Cu processed by a single HPT revolution was also reported by Jamalian et al. [45]. However, they did not apply any initial annealing and thus started the processing at the original microhardness of about 115 HV.

## 5. Conclusions

The aim of this study was to find out conditions of high pressure torsion (HPT) processing at room and cryogenic temperatures, which would yield comparable results as regards (sub)structure development within the original annealed Cu, and then compare such specimens from the viewpoints of mechanical properties, i.e., microhardness, and electric properties. The results revealed that cryo-HPT was highly efficient; 180° revolution imparted structure refinement down to the average grain size value of 7.2 µm, which was comparable to the refinement acquired after 360° revolution of room temperature (RT) HPT. Both the specimens featured increased electric conductivity when compared to the original Cu; the 180° cryo-HPT specimen featured the highest observed conductivity of 59.66 MS·m^−1^. Interestingly, the cryo-HPT specimen also featured the highest observed microhardness (average value of 118.2 HV0.2), and its homogeneity across the cross-section of the specimen was higher than that observed within the RT HPT specimen, too. These differences could be attributed primarily to differences in substructure development–due to the aggravated plastic flow, the 180° cryo-HPT specimen featured more or less randomly oriented grains of equiaxed shapes with a significant occurrence of twins in both the micro and nano scales, whereas visibly deformed grains with inhomogeneous substructure development, i.e., dislocations density, and negligible occurrence of twining was observed after 360° rotation of RT HPT.

## Data Availability

The original data supporting the research is not publicly available, but a portion of the data that is not confidential is available on request from the corresponding author.
